# Potential common mechanisms between primary Sjögren’s syndrome and Hashimoto’s thyroiditis: a public databases-based study

**DOI:** 10.3389/fgene.2025.1520332

**Published:** 2025-04-29

**Authors:** Yanjun Lin, Shupin Tang, Yan Lin, Rihui Wang, Yifeng Xing, Zonghe Xu, Yan Li, Qingxia Fang, Wenwei Wei, Dong Wu, Jiang Chen

**Affiliations:** ^1^ Fujian Key Laboratory of Oral Diseases, School and Hospital of Stomatology, Fujian Medical University, Fuzhou, Fujian, China; ^2^ Department of Otorhinolaryngology-Head and Neck Surgery, the First Affiliated Hospital, Fujian Medical University, Fuzhou, Fujian, China; ^3^ Department of Oral and Maxillofacial Surgery, the First Affiliated Hospital, Fujian Medical University, Fuzhou, Fujian, China; ^4^ Research Center of Dental and Craniofacial Implants, School and Hospital of Stomatology, Fujian Medical University, Fuzhou, Fujian, China; ^5^ Department of Oral Implantology, School and Hospital of Stomatology, Fujian Medical University, Fuzhou, Fujian, China

**Keywords:** primary Sjögren’s syndrome, Hashimoto’s thyroiditis, common mechanisms, cross-talk genes, hub genes

## Abstract

**Objective:**

Primary Sjögren’s syndrome (pSS) and Hashimoto thyroiditis (HT) can occur in the same patient population, but the mechanism of co-occurrence remains unknown. This study aims to explore the underlying mechanism.

**Methods:**

We screened differentially expressed genes (DEGs) in the pSS and HT-related transcriptomic microarrays. Based on KEGG, PID, Reactome, and BioCarta enrichment analysis, pathway annotations were performed. A PPI network was developed using STRING. Betweenness, BottleNeck, MNC, Radiality EPC, and Stress topological analyses were performed to identify hub genes. Then, we used two more datasets to validate the key genes. Immune infiltration landscape of pSS and HT were profiled based on CIBERSORT, Xcell, MCPCounter, and EPIC. Correlation between T/B cells and key genes was performed. Single gene GSEA analysis was performed to further explore enriched pathways of key genes. Finally, we predicted the drugs of key genes and the cross-talk genes targeted in the protein domain.

**Results:**

A total of 93 cross-talk genes were found. These genes were mainly related to the immune system. STAT1, CD8A, and PTPRC were identified as hub genes using six topological methods. STAT1 and PTPRC are considered key genes after *in silico* validation. STAT1 and PTPRC were linked to CD8^+^ Tcm and other immune cells in the pSS and HT dataset. GSEA analysis showed that STAT1 and PTPRC may play a role in pSS and HT through several pathways, including IFNγ response, IFNα response, allograft rejection, E2F targets, complement, G2M checkpoint, IL6-JAK-STAT3 signaling, KRAS signaling up, IL2-STAT5 signaling, IL6-JAK-STAT3-signaling, and inflammatory response. Guttiferone K and picoplatin may be the candidate drugs for the treatment of pSS and HT. Cross-talk genes were mainly enriched in IGc1, MHCIIα and SCY.

**Conclusion:**

We analysed databases and gene expression data for pSS and HT. We identified two genes (STAT1, PTPRC) as potential biomarkers of disease activity in pSS and HT. We also gained new insights into the cellular and molecular mechanisms associated with pSS and HT. Based on the key genes and cross-talk genes, we predicted potential drugs and protein domains for pSS and HT.

## Introduction

Sjogren’s syndrome (SS) is a chronic systemic immune-mediated inflammatory rheumatic disease involving lymphocyte infiltration into salivary and lacrimal glands, resulting in gland dysfunction and destruction (Zhan et al., 2023). The principal indications of SS are corneal staining (+), tear secretion of ≤5 mm/5 min, salivary flow rate of ≤1.5 mL/15 min, lymphocyte infiltration index of the lacrimal gland of ≥1 foci/4 mm^2^, and the presence of positive serum anti-SSA and anti-SSB antibodies (Shiboski et al., 2016). The prevailing treatment approach of SS is using immunomodulatory therapies to alleviate sicca symptoms and control inflammation (Vivino et al., 2019). SS could occur alone as a primary Sjögren’s syndrome (pSS) or in conjunction with another systemic autoimmune disease, such as systemic lupus erythematosus or Hashimoto’s thyroiditis, which was called secondary Sjögren’s syndrome (sSS) (Negrini et al., 2022). In general populations, the prevalence of pSS has been estimated to lie between 0.0024% and 4.8%. Most pSS patients are women, with a male-to-female ratio of approximately 1:14 (Beydon et al., 2024).

Hashimoto’s thyroiditis (HT) is a chronic systemic immune-mediated inflammatory rheumatic disease, which is regarded as the most prevalent autoimmune thyroid disease (AITD). The pathological hallmark features are lymphoplasmacytic infiltration, lymphoid follicle formation with germinal centers, and parenchymal atrophy, which may cause gland dysfunction (Yuan et al., 2023). The diagnosis of HT is mainly based on clinical symptoms of hypothyroidism and the presence of serum antibodies against thyroid peroxidase (TPOAbs) (Klubo-Gwiezdzinska and Wartofsky, 2022). The mainstay of treatment is the management of hypothyroidism with thyroxine substitution therapy. The prevalence of HT increases with age, particularly in patients diagnosed with other autoimmune conditions, including myasthenia gravis (Song et al., 2019), systemic sclerosis (Yao et al., 2019), and SS (Bliddal et al., 2017). The estimated incidence of HT is 3–15 cases per 10,000 people, with a female-to-male predominance ratio of 7–10:1 (Ralli et al., 2020).

Despite unclear etiology, pSS and HT are multifactorial diseases involving genetic predispositions epigenetics, and environmental risk factors (Zhang et al., 2023). A correlation between HT and pSS was identified, with a higher prevalence of HT observed among pSS patients (6.26%) compared to the general population (1%–2%) (Zeher et al., 2009). The most common pathophysiological features of pSS and HT are lymphocyte infiltration of glandular tissue and glandular dysfunction. The clinical manifestations of SS exhibit slight differences when HT is coexisting. Some scholars have proposed that pSS and HT share a common physiopathological mechanism. Despite their distinct nomenclature, the coexistence of these conditions should be interpreted as polyautoimmunity (Anaya et al., 2019).

**FIGURE 1:**
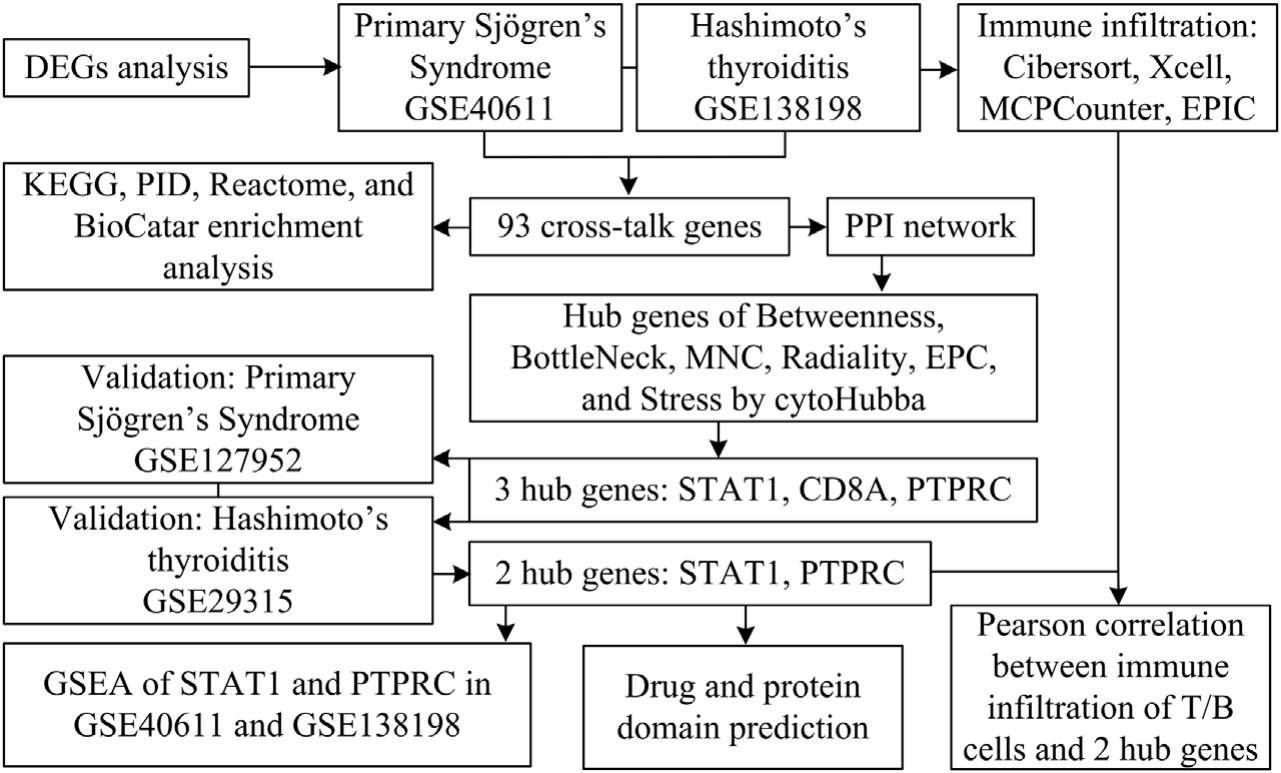
Study flowchart.

There are numerous parallels between pSS and HT with regard to demographic characteristics and pathophysiology. However, few articles have reported on the pathogenesis of the co-occurrence of pSS and HT. Thus, we aimed to explore the common mechanism of this comorbidity using a variety of bioinformatic methods based on available public data. Figure 1 shows the flowchart for the research.

## Methods

### Dataset information and the cross-talk genes

The salivary glands and thyroid glands gene expression matrices of GSE40611 (Horvath et al., 2012) (17 pSS patients, 18 controls) and GSE138198 (Subhi et al., 2020) (12 HT patients, three controls) were collected from Gene Expression Omnibus (GEO) (https://www.ncbi.nlm.nih.gov/). All data of gene expression were subjected to log2 transformation. We downloaded the relevant annotation documents from the Gemma (https://gemma.msl.ubc.ca/). Annotation documents mapped microarray probes to gene symbols. The differentially expressed genes (DEGs) were determined between cases and control using the limma R package. The FDR <0.05 and |Fold Change| >1.5 were considered significant. A Venn diagram was employed to derive the differentially expressed cross-talk genes (Bardou et al., 2014).

### Functional enrichment analysis

We downloaded the subset from the Molecular Signatures Database (https://www.gsea-msigdb.org/gsea/msigdb/index.jsp) as a mapping background and used the R package clusterProfile to obtain the gene set enrichment results. Functional enrichment analysis was performed with common DEGs of GSE40611 and GE138198 based on KEGG, PID, Reactome, and BioCatar gene sets (Li et al., 2019; Dong et al., 2023; Schaefer et al., 2009). The minimum gene set was set to five and the maximum gene set was set to 5,000. The cutoff values for p- and FDR-values were set at 0.05.

### Protein-protein interaction network construction

Protein-protein interaction network of potential crosstalk genes was constructed using the STRING database (https://cn.string-db.org/) with a confidence score threshold set to 0.4 and visualised in Cytoscape software (V3.9.1). The genes mentioned above were further analyzed using the CytoHubba plugin. Six topological analysis algorithms were selected: Betweenness, BottleNeck, maximum neighbour component (MNC), Radiality, edge percolated component (EPC), and Stress (Chin et al., 2014; Tan et al., 2024). The top 10 genes after six algorithms were intersected to obtain hub shared genes.

### Identification and validation of hub genes

GSE127952 containing pSS patients and GSE29315 containing HT patients were examined to validate the hub genes, presented using violin plots. ROC curves were used to explain the relation existing between the sensibility and the specificity of hub genes (Zeng et al., 2023).

### Immune infiltration analysis and correlation analysis

We assessed the levels of immune cells in pSS and HT patients using CIBERSORT, XCell, MCPCounter, and EPIC algorithm in the R package in GSE40611 and GSE138198 datasets (Yi et al., 2022). A Pearson correlation analysis was conducted between the T/B cells and the hub-shared genes using the “ggplot2” package.

### Gene set enrichment analysis

To further explore the potential function of the selected hub shared genes in pSS and HT, GSEA for the single hub-shared genes was performed (Subramanian et al., 2005). In the dataset GSE40611 and GSE138198, samples were divided into high expression group ( ≥ 50%) and low expression group (<50%) according to the expression level of hub shared genes. The R package “clusterprofiler” was utilized to conduct GSEA (Yu et al., 2012). The hallmark gene sets in Molecular Signatures Database (MSigDB) was selected as the reference gene set (Liberzon et al., 2015). *p-value* < 0.0 was chosen as the cut-off criteria.

### Drug and protein domain prediction

The DGIdb database, accessible via the URL https://www.dgidb.org/, is a drug-gene interaction database that collates data from diverse sources, including databases, articles, and websites, on drug and gene interactions. The interaction scores rely on evidence score, relative drug specificity, and relative gene specificity. Evidence score = publication count + source count. Relative drug specificity = average known gene partners for all drugs/known gene partners for drug d. Relative gene specificity = average known drug partners for all genes/known drug partners for gene g (Freshour et al., 2021). The database identifies potential drug candidates for pSS and HT based on hub shared genes. The Funrich V3.1.3 software (http://www.funrich.org/) was used to perform protein domain enrichment analysis on the cross-talk proteins. This software is an open-access, stand-alone tool for functional enrichment (Fonseka et al., 2021). *p* < 0.05 is set as the threshold.

## Results

### Identification of cross-talk genes in pSS and HT

**FIGURE 2:**
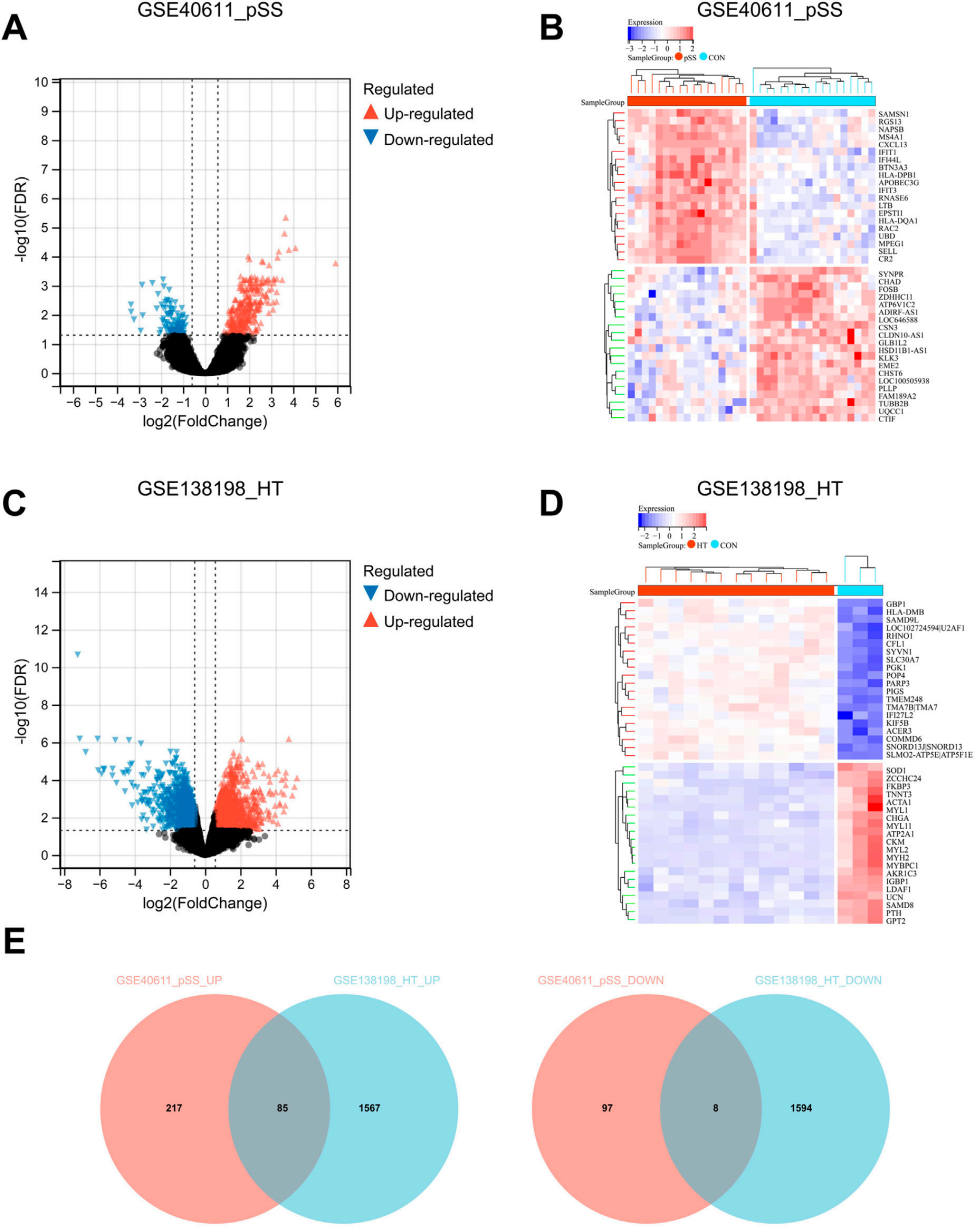
Identification of cross-talk genes in pSS and HT. **(A)** Volcano plot shows up- (red) and down- (blue) regulated DEGs in GSE40611. **(B)** Heatmap plot GSE40611 shows TOP20 up- and downregulated DEGs. **(C)** Volcano plot shows up- (red) and down- (blue) regulated DEGs in GSE138198. **(D)** Heatmap plot GSE138198 shows TOP20 up- and downregulated DEGs. **(E)** Cross-talk up- and downregulated genes in pSS and HT are plotted using Venn after the intersection of GSE40611 and GSE138198. In volcano plots, FDR <0.05 and |Fold Change| >1.5. The samples in heatmap plots are grouped and clustered according to disease and control using the Euclidean method with FDR <0.05.

To investigate the cross-talk genes underlying the occurrence and progression of pSS and HT, we selected two independent datasets of pSS (GSE40611) and HT (GSE138198). The characteristics of the involved transcriptomic microarray datasets were shown (Table 1), including GSE ID, types of diseases, samples, and detection platforms. The GSE40611 dataset contained 407 differentially expressed genes, including 302 upregulated and 105 downregulated genes. The GSE138198 dataset contained 3,254 differentially expressed genes, including 1,652 upregulated and 1,602 downregulated genes. We showed the DEGs using the volcano plots and the clustering effect of samples of cases or controls using the heatmap plots (Figures 2A–D), which indicated apparent differences in marker expression between the objects. Taking the intersection of the DEGs in GSE40611 and GSE138198, we identified 93 cross-talk genes (85 upregulated and eight downregulated) (Figure 2E).

**TABLE 1:** Characteristics of the involved transcriptomic microarray datasets.

GSE ID	Case	Control	Sample	Platform	Year	PMID
GSE40611	17 pSS	18 CON	Parotid	GPL570	2012	23116360
GSE138198	12 HT	3 CON	Thyroid	GPL6244	2020	32603365

### Functional annotation of cross-talk genes

**FIGURE 3:**
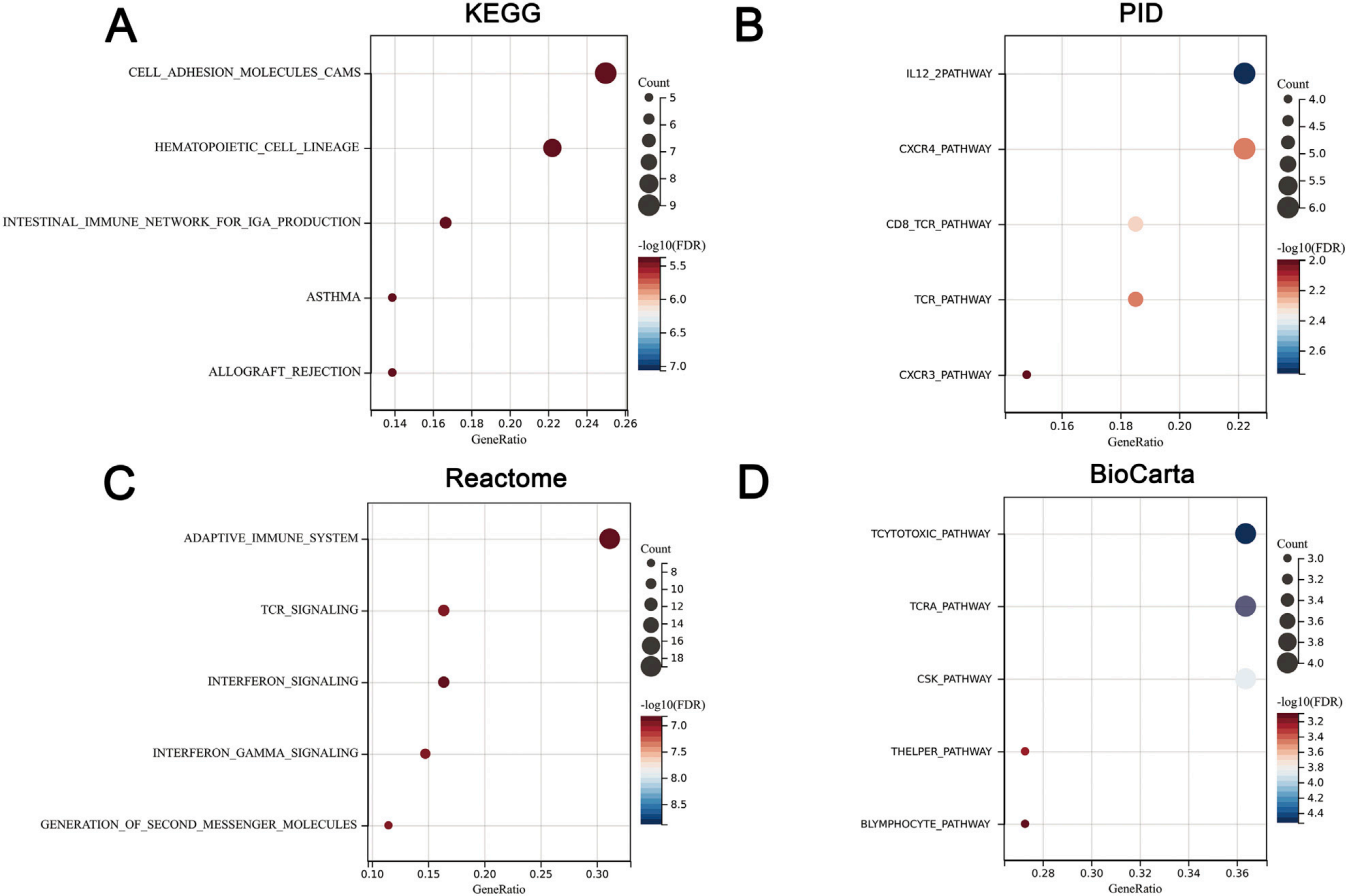
Functional annotation of cross-talk genes. **(A)** KEGG enrichment. **(B)** PID enrichment. **(C)** Reactome enrichment. **(D)** BioCatar enrichment. The X-axis represents gene ratio while the Y-axis represents TOP5 enriched pathways. The size of the dots represents the number of genes, and the color of the dots represents the magnitude of the FDR. FDR <0.05.

We uploaded the 93 DEGs to perform the KEGG, PID, Reactome, and BioCatar analysis. The outcomes of KEGG enrichment analysis revealed that cell adhesion molecules (CAMs), hematopoietic cell lineage, intestinal immune network for IgA production, asthma, and allograft rejection were involved (Figure 3A). The PID enrichment analysis revealed that entries such as IL12 2pathway, CXCR4 pathway, CD8 TCR pathway, and CXCR3 pathway participated in the pathogenesis (Figure 3B). The Top five terms of Reactome enrichment analysis indicated that adaptive immune system, TCR signaling, interferon signaling, interferonγ signaling, and generation of second messenger molecules were involved (Figure 3C). The outcomes of BioCatar enrichment analysis revealed that T cytotoxic pathway, TCRa pathway, CSK pathway, T helper pathway, and B lymphocyte pathway were involved (Figure 3D).

### Hub genes screening

**FIGURE 4:**
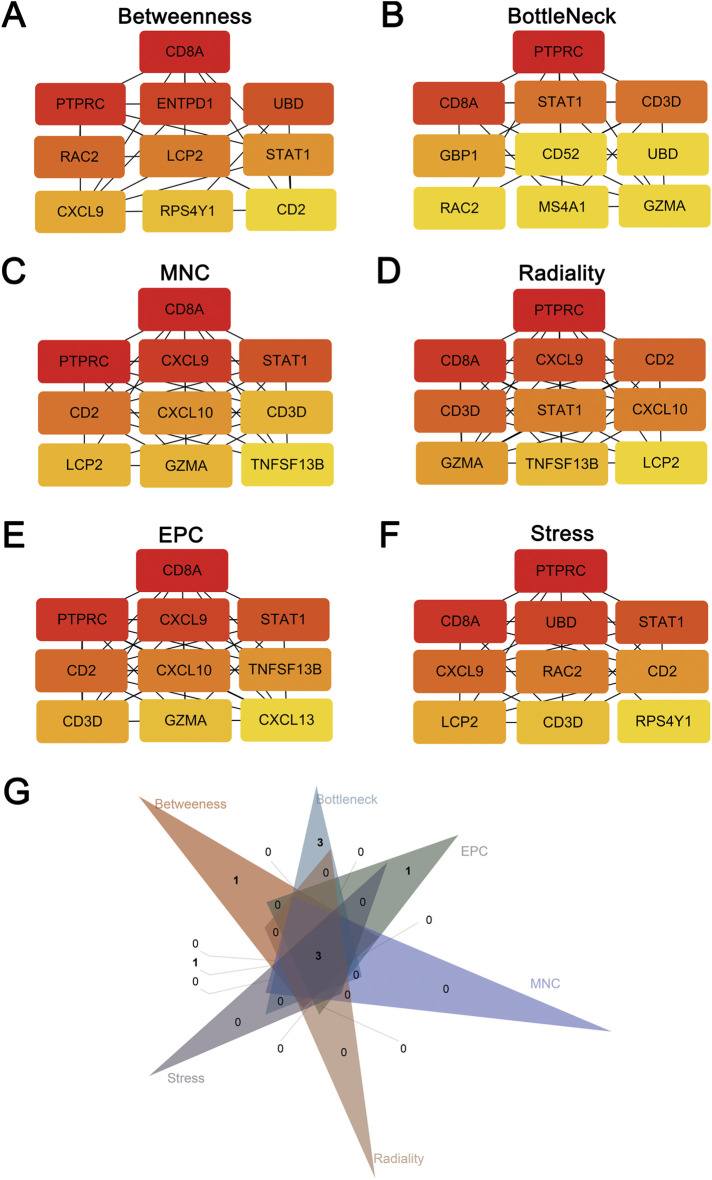
Hub genes screening after protein-protein interaction analysis. **(A)** TOP10 hub genes screened by the Betweenness method. **(B)** TOP10 hub genes screened by the BottleNeck method. **(C)** TOP10 hub genes screened by the MNC method. **(D)** TOP10 hub genes screened by the Radiality method. **(E)** TOP10 hub genes screened by the EPC method. **(F)** TOP10 hub genes screened by the Stress method. **(G)** Three hub genes (STAT1, CD8A, PTPRC) after intersection of six screening methods using Venn plot.

The cross-talk genes of GSE40611 and GSE138198 were subjected to a protein-protein interaction analysis to obtain an interworking network, which was subsequently imported into Cytoscape’s Cytohubba plug-in. The results of the six topological analyses (Betweenness, BottleNeck, MNC, Radiality, EPC, and Stress) are presented in Figures 4A–F. The common three core genes for the six analyses were STAT1, CD8A, and PTPRC (Fig. G).

### Hub genes *in silico* validation

**FIGURE 5:**
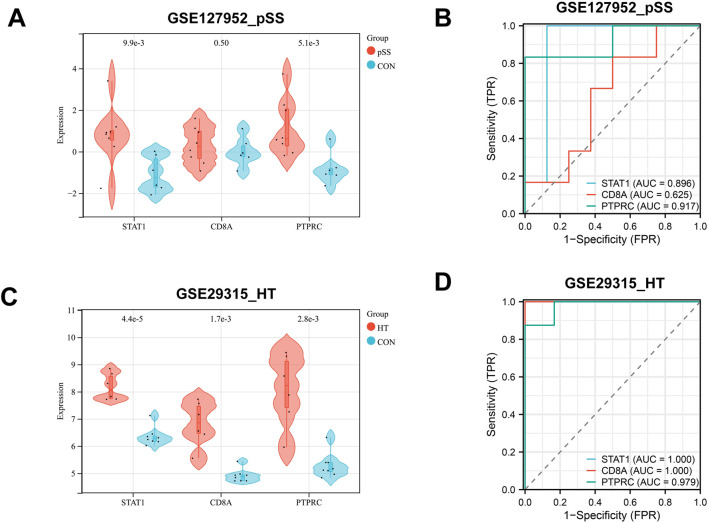
Hub genes *in silico* validation. **(A)** STAT1, CD8A, and PTPRC expressions are validated in another pSS dataset of GSE127952. **(B)** ROC curve of three hub genes in GSE127952. **(C)** STAT1, CD8A, and PTPRC expressions are validated in another HT dataset of GSE29315. **(D)** ROC curve of three hub genes in GSE29315. ROC curves reflect the relationship between sensitivity and specificity. The closer the AUC is to 1, the better the diagnostic effect of the variable in predicting the outcome.

The expression levels of shared hub genes were further validated in the two datasets. The expression of STAT1 and PTPRC in GSE127952 (pSS) was significantly higher than that in the control group (*p* < 0.05) (Figure 5A). The AUC values of hub genes in GSE127952 (pSS) were 0.896 (STAT1), 0.625 (CD8A), and 0.917 (PTPRC) (Figure 5B). The expression of all the hub genes (STAT1, CD8A, and PTPRC) in GSE29315 (HT) was significantly higher than that in the control group (*p* < 0.05) (Figure 5C). The AUC values of hub genes in GSE29315 (HT) were 1.000 (STAT1), 1.000 (CD8A), and 0.979 (PTPRC) (Figure 5D). Only two key genes, STAT1 and PTPRC, have an AUC value of >0.85 in both datasets.

### Immune infiltration landscape and correlation between T/B cells and key genes

**FIGURE 6:**
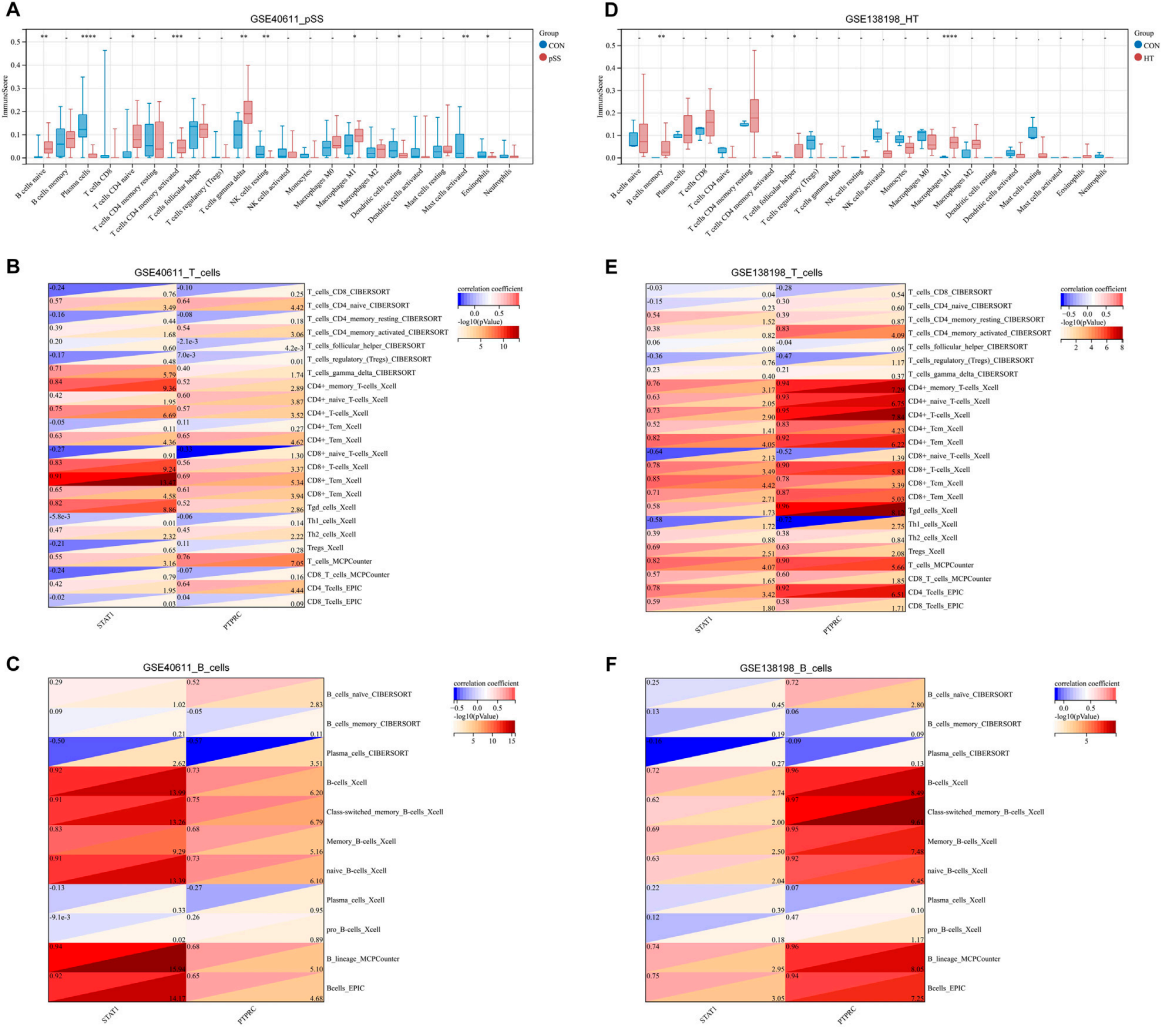
Immune infiltration landscape and correlation between T/B cells and key genes. **(A)** Immune infiltration of pSS using the CIBERSORT method. **(B)** Immune infiltration of HT using the CIBERSORT method. **(C)** Correlation between T cells and STAT1/PTPRC in pSS. **(D)** Correlation between T cells and STAT1/PTPRC in HT. **(E)** Correlation between B cells and STAT1/PTPRC in pSS. **(F)** Correlation between B cells and STAT1/PTPRC in HT. Correlation analysis is based on Pearson’s method. A red correlation coefficient indicates a positive relationship between T/B cells and STAT1/PTPRC; a blue one, a negative one. The threshold criteria are *p* < 0.05 and -log10 (*p*-value) < 1.3.

It remains unclear whether pSS and HT share common pathological mechanisms or similar immune microenvironments. With GSE440611 and GSE138198 datasets, we assess the extent of infiltration of 22 immune cell types using the Cibersort algorithm. pSS and HT demonstrated different immune infiltration patterns. Figure 6A showed that the abundance of B cells naïve, plasma cells, T cells CD4 naïve, T cells CD4 memory activated, T cells gamma delta, NK cells resting, macrophages M1, dendritic cells resting, mast cells activated, and eosinophils are significantly different between cases and controls. Figure 6B showed that B cells memory, T cells CD4 memory activated, T cells follicular helper, and macrophages M1 are significantly different between cases and controls. pSS and HT shared only two entries, T cells CD4 memory activated and macrophages M1. STAT1 expression in the pSS and HT dataset was positively associated with CD8^+^ Tcm (Xcell) with a correlation coefficient of >0.8. STAT1 expression in the pSS and HT dataset was positively associated with CD4^+^ memory T cells (Xcell), CD4^+^ T cells (Xcell), CD4^+^ Tcm (Xcell), CD8^+^ T cells (Xcell), CD8^+^ Tem (Xcell), B cells (Xcell), class-switched B cells (Xcell), memory B cells (Xcell), naïve B cells (Xcell), B lineage (MCPCounter), and B cells (EPC) with a correlation coefficient of <0.8 and >0.6. PTPRC expression in the pSS and HT dataset was positively associated with CD4^+^ Tem (Xcell), CD8^+^ Tcm (Xcell), CD8^+^ Tem (Xcell), T cells (MCPCounter), CD4 T cells (EPIC), B cells (Xcell), class-switched B cells (Xcell), memory B cells (Xcell), naïve B cells (Xcell), B lineage (MCPCounter), and B cells (EPC) with a correlation coefficient of <0.8 and >0.6 (Figures 6C–F). It indicated that STAT1 is strongly associated with CD8^+^ Tcm in the pathogenesis of pSS and HT.

### Single-gene GSEA of key genes

**FIGURE 7:**
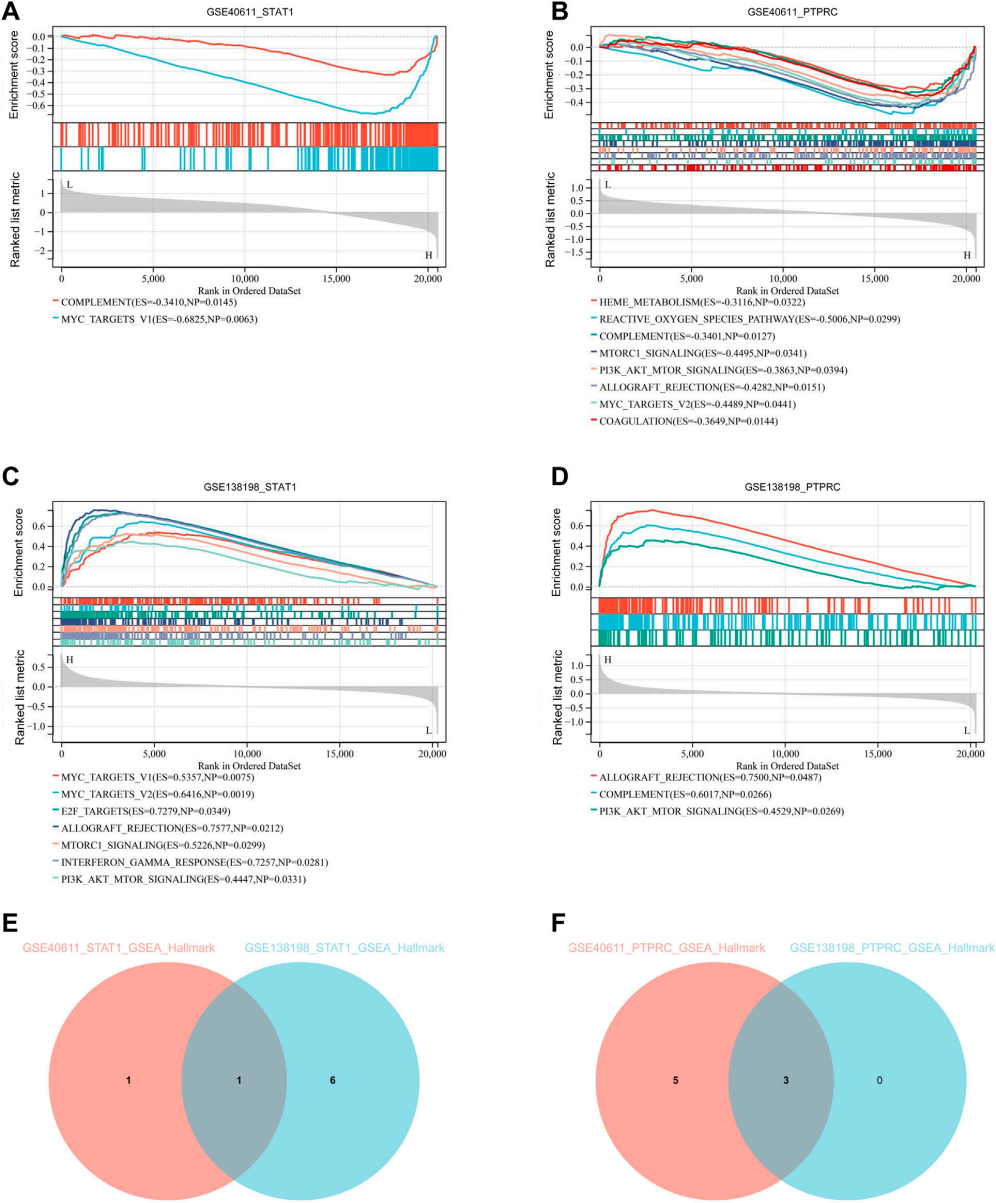
Single-gene GSEA of key genes. **(A)** Single-gene GSEA of STAT1 in GSE40611 shows two entries. **(B)** Single-gene GSEA of PTPRC in GSE40611 shows eight entries. **(C)** Single-gene GSEA of STAT1 in GSE138198 seven entries. **(D)** Single-gene GSEA of PTPRC in GSE1138198 three entries. **(E)** Intersection of GSEA enriched entries for STAT1 in pSS and HT shows one entry. **(F)** Intersection of GSEA enriched entries for PTPRC in pSS and HT shows three entries. The cut-off criterion is *p* < 0.05.

We performed single-gene GSEA analyses of two key genes, STAT1 and PTPCR. The results showed that within GSE40611, there are two entries for STAT1 and eight for PTPCR based on the Hallmark database. Within the GSE138198 are seven entries for STAT1 and three for PTPCR (Figures 7A–D). Enriched entries of STAT1 after the intersection of two datasets showed that MYC_TARGETS_V1 was the common entry (Figure 7E). Taking the intersection of the enriched entries of PTPCR from two datasets showed that complement, PI3K-AKT-mTOR signaling, and allograft rejection were shared entries (Figure 7F). The results suggest that STAT1 and PTPRC may play a role in the disease process of pSS and HT through these shared entries.

### Drug prediction of key genes and prediction of cross-talk genes targeted protein domain

**FIGURE 8:**
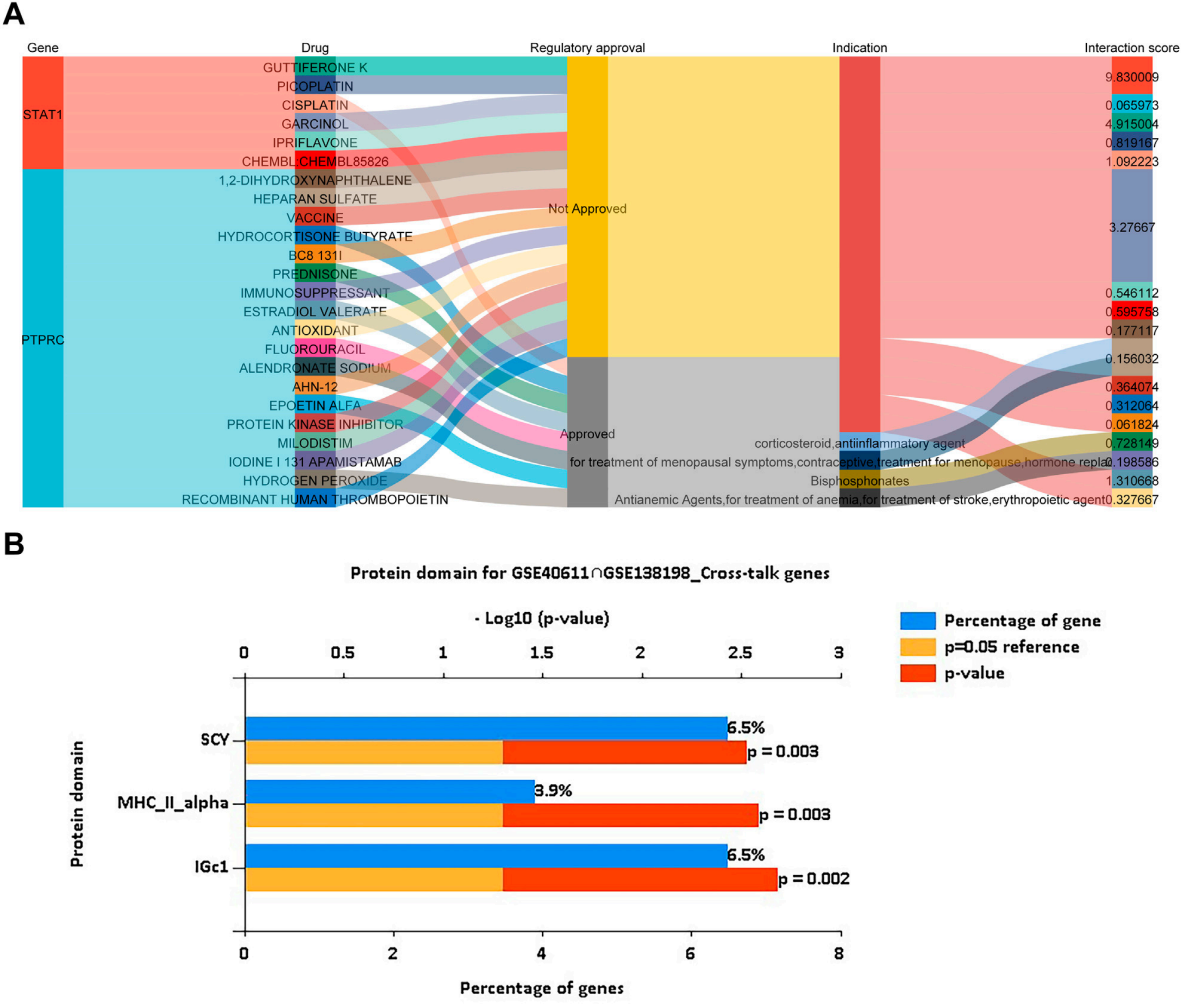
Drug prediction of key genes and prediction of cross-talk genes targeted protein domain. **(A)** Drug prediction of STAT1 and PTPRC shows the flow path of gene, drug, regulatory approval, indication, and interaction score using a Sankey plot. **(B)** Protein domain prediction of cross-talk genes of pSS and HT indicates IGc1 (6.5% genes), MHCIIα (3.9% genes) and SCY (6.5% genes), using a bar plot. The cut-off criteria are *p* < 0.05.

Using the DGIdb database, we analyzed potential drugs for pSS and HT. 24 predicted drugs corresponded to two key genes. The Sankey diagram revealed the relationships between gene, drug, regulatory approval, indication, and interaction score (Figure 8A). Among the predicted drugs, two drugs (guttiferone K and picoplatin) had the highest interaction score, proving that the candidate drugs for the treatment of pSS and HT. It was postulated that the majority of potential pharmaceutical agents might interact with the key gene in ways that were either unknown or inhibitory. Protein domain enrichment analysis showed that the DEGs were mainly enriched in IGc1, MHCIIα, and SCY (*p* < 0.05). This suggests that these structural domains may be the target of drug action (Figure 8B).

## Discussion

Patients presenting with pSS showed an incidence of HT that was three-to sixfold higher than in the general population (Biró et al., 2006). Another study of 170 patients with Hashimoto’s thyroiditis revealed that 17% of them also had Sjögren’s syndrome (Zeher et al., 2009). The coexistence of these two diseases is frequent and suggests a common genetic predisposition between pSS and HT (Foster et al., 1993; Jara et al., 2007). From an immunological perspective, thyroid and salivary glands may share the same antigens, which attract similar lymphocytic infiltration to attack the secretory structures. The antihuman thyroglobulin in patients with pSS and HT exhibited significant overlap within a single region of human thyroglobulin, indicating that the shared pathogenetic mechanisms underlying pSS and HT may involve the specific region (Bouanani et al., 1991). Other potential explanations for the observed correlation between pSS and HT include a shared genetic predisposition or environmental factors. In pSS patients, periodic examination of thyroid function is essential to enable prompt implementation of appropriate treatment in the event of hypothyroidism. It is similarly crucial for individuals with HT to undergo regular assessment of their salivary gland function, in order to facilitate prompt implementation of appropriate therapeutic measures in the event of salivary dysfunction or xerostomia. Autoimmune polyglandular syndrome (APS) represents a series of autoimmune disorders characterized by the co-occurrence of multiple gland dysfunction. APS can be divided into four types and may exacerbate the course of autoimmune rheumatic diseases. APS-2 and APS-3 are the most prevalent forms. APS-2 encompasses the coexistence of HT, coeliac disease, and rheumatoid arthritis (RA), among other conditions. APS-3 is considered to be a variant of APS-2. In APS-3, RA, systemic lupus erythematosus (SLE), and pSS may coexist with HT, type 1 diabetes mellitus, and hypogonadism or other endocrine disorders. The exacerbation of metabolic disturbances observed in the course of rheumatic diseases may be attributed to undiagnosed endocrine diseases, which can lead to ineffective treatment of rheumatic diseases (Jankowska et al., 2023). Both pSS and HT are defined as systematic disorders of immune homeostasis. The imbalanced activation and pathological immune responses affecting both the cellular and humoral components of immunity contribute to organ injury. pSS affects exocrine glands, which can lead to chronic lymphocytic sialadenitis and autoimmune epithelitis. HT affects endocrine glands, which can lead to chronic lymphocytic thyroiditis. Therefore, it is hypothesized that the salivary glands and thyroid suffer from the same kind of autoimmune disturbance in pSS and HT. Subsequently, public data was utilized to identify the common pattern of genes, pathways and immune cells.

PTPRC and STAT1 served as two of the TOP 10 genes based on the PPI results of the shared DEGs. The ROC curve also indicated the predominant diagnostic value of STAT1 and PTPRC for both pSS and HT. The levels of STAT1 in T and B cells are found to correlate with the CD169 on monocytes. The high expressions of STAT1 and IRF9 within B cells of pSS are significantly associated with hypergammaglobulinemia and anti-SSA/SSB autoantibodies. Elevated STAT1 level, as a subgroup biomarker, is also observed in extra glandular manifestations (Ritter et al., 2024). An antibody that specifically recognizes the phosphorylated tyrosine residue in amino acid position 701 enables the detection of activated STAT1 dimers in numerous germinal macrophages and infiltrating lymphocytes of HT patients (Staab et al., 2012). Hyperexpression of HLA I is a defining feature of HT, whose colocalization with STAT1 was found. The colocalization of HLA I and STAT1 indicates an intracellular, antiviral host response. Thyroid cells express coxsackie and adenovirus receptor, thus providing a link between viral infection and HT (Weider et al., 2020). PTPRC, also known as CD45, represents approximately 10% of the surface antigens of T and B lymphocytes. It plays a regulatory role in activating T and B cells which controls immune function primarily by modulating cytokine responses and TCR signaling (Lin et al., 2022). PTPRC has been shown to inhibit the JAK-STAT signaling pathway, which mediates the activation of various cytokines via intracellular signal transduction. The imbalance of the JAK-STAT signaling pathway contributes to numerous autoimmune diseases including HT and pSS. For instance, the percentage of CD45^+^HLA-DR^+^ cells positively correlates with the clinical severity of Sjögren’s syndrome keratoconjunctivitis sicca and negatively correlates with the conjunctival goblet cell density (Pflugfelder et al., 2018). A reduction in CD45RAB transcript levels is observed in conjunction with an age-related transition from a naive to a memory/late-differentiated T cell CD45R mRNA signature, which is associated with thyroid hormone status in HT patients. This suggests that PTPRC may play a role in the pathogenesis of HT (Tokić et al., 2017).

The single-gene GSEA of STAT1 and PTPCR indicated that MYC targets V1, complement, PI3K-AKT-mTOR signaling, and allograft rejection were involved in the common pathogenesis of pSS and HT. MYC targets V1 are a subgroup of genes regulated by MYC - version 1, including KRAS, MYC, and HDAC2. Mutations in the KRAS gene can also produce autoimmune-like lymphoproliferative syndrome (ALPS)-like manifestations, like pSS and HT. For instance, Ras-associated autoimmune lymphoproliferative disorder (RALD) is a sporadic primary immunodeficiency disease caused by somatic mutations in the KRAS and NRAS genes, which result in ALPS-like manifestations (Neven et al., 2021). In the case of pSS, the expressions of HDAC two are relatively low in the salivary glands (Yang et al., 2024). PIK3CA, AKT1, mTOR, and MYC are upregulated in activated CD4 T cells of patients with pSS (Chen et al., 2024). Compared with the regions of normal surrounding thyroid tissue, a notable increase in the expression of phosphorylated Akt, Akt1, and Akt2 was observed in the regions of HT and thyroid cancer (Larson et al., 2007). Primary thyroid high-grade B-cell lymphoma with MYC rearrangements can be observed in HT (Tariq and Zahra, 2022). The term complement is now regarded as a pivotal component of innate immunity, safeguarding the host against pathogens, orchestrating the multifaceted processes of the inflammatory response, integrating innate and adaptive immune responses, and crosstalking with other effector systems (Thorgersen et al., 2019). The term allograft rejection indicate that the autoimmune responses of pSS and HT are similar to allograft rejection, could participate in targeted tissue damage (Jeon and Choi, 2016).

The results indicated that pSS and HT did not have the same causative immune cells. The common entries of pSS and HT consisted of T cells and B cells based on different immune infiltration analysis. The expression of CD45RA and CCR7 allows for the classification of CD4^+^ T cells into three populations: T naive (CD45RA^+^CCR7^+^), Tcm (CD45RA^−^CCR7^+^) and Tem (CD45RA^−^CCR7^-^). Similarly, CD8^+^ T cells can be classified into four populations: T naive (CD45RA^+^CCR7^+^), Tem (CD45RA^−^CCR7^-^), Tcm (CD45RA^−^CCR7^+^) and TemRA (CD45RA^+^CCR7^-^) (Lanio et al., 2009). STAT1 is strongly associated with CD8^+^ Tcm in the pathogenesis of pSS and HT. CD4^+^ T cells, CD8^+^ T cells, and macrophages are abnormally increased in patients with HT and Graves’ disease. The differentially expressed genes of these cells are significantly involved in signaling pathways, including Th1 and Th2 cell differentiation, Th17 cell differentiation, cytokine-cytokine receptor interaction, and NF-kappa B signaling pathway. Moreover, in HT, CD4^+^ T cells interact with macrophages via the IL16-CCR5/FGF10-FGFR1/CXCL13-CXCR3 axis, and macrophages interact with CD8^+^ T cells via the CD70^−^CD27 axis, thereby activating the T-cell receptor signaling pathway and NF-kappa B signaling pathway (Zheng et al., 2022). After stimulating PBMCs with HT exosomes, CD11c^+^TLR2^+^/TLR3^+^ and CD4^+^IFN-γ^+^Th1/IL-17A^+^Th17 cell percentages are significantly elevated, and CD4^+^CD25^+^Foxp3^+^ Treg cell percentage is significantly decreased. HT exosomes induced increase IL-17A and IFN-γ production, whereas IL-10 production is suppressed (Cui et al., 2019). Both antigens, TPO and Tg, are recognized by CD8^+^ T cells and are involved in the thyroid destruction process leading to clinical disease manifestation (Ehlers et al., 2012). In certain autoimmune disorders, autoreactive CD4^+^ T cells frequently exhibit a Th17 or mixed Th1-Th17 phenotype. The affinity of autoreactive CD4^+^ T cells is typically lower than that of CD4^+^ T cells that respond to foreign antigens, as high-affinity clones are often eliminated or differentiate into regulatory T cells during development (Künzli and Masopust, 2023). A noteworthy increase in the number of CD8^+^ T cells was observed in patients with pSS. A greater proportion of peripheral blood GZMK^+^CXCR6^+^CD8^+^ T cells exhibited shared clones with CD69^+^CD103^−^CD8^+^ tissue-resident memory T (Trm) cells in the labial glands of patients with pSS. CD69^+^CD103^−^CD8^+^ Trm cells, which are characterised by high GZMK expression, were observed to exhibit greater activity and cytotoxicity than CD103^+^ cells. IL-15 was significantly elevated in pSS plasma and demonstrated the capacity to promote the differentiation of CD8^+^ T cells into GZMK^+^CXCR6^+^CD8^+^ T cells in a STAT5-dependent manner (Xu et al., 2023). CD103^+^CD8^+^ Trm cells expanded in the salivary glands of pSS, contributing to the secretion of granzyme-B and interferon-γ (Mauro et al., 2024). CD4^+^ T cells of pSS patients contributed to autoantibody production and lymphocytic infiltration through multiple cytokines (Yao et al., 2021). Salivary gland ductal cells of pSS are typically encased in a CD4^+^ T cell and B cell infiltration. B cell infiltration within the ducts can precipitate the formation of lymphoepithelial lesions, including basal ductal cell hyperplasia (Verstappen et al., 2021). Various pathogenic B cell subsets, like FcRL4 B cell, age-associated B cell, transitional B cell, marginal zone B cell, memory B cell and plasma cell, contribute to disease progression in pSS. Regulatory B cells, like IL-10^+^ Breg, GrB^+^ Breg, IL-35^+^ Breg and regulatory plasma cell attenuate disease activity (Du et al., 2021). Aberrant functions of T cell subsets play a pivotal role in disrupting immune homeostasis and initiating an autoimmune cascade against thyroid tissues. It is also hypothesised that one of the earliest stages in the development of HT is a functional alteration of B cells, which results in the formation of autoantibodies (Ralli et al., 2020). Some functional defects of Breg may be the cause of HT (Kang et al., 2021).

Guttiferones K and picoplatin are the potential drug for pSS and HT. Direct interaction of guttiferones K with the transcription factor STAT-1 as a likely mechanism of their inhibitory effect on cytokine signaling pathways (Masullo et al., 2014). Picoplatin, a cytotoxic platinum-based compound, is currently under clinical development for treating patients suffering from solid tumors (Wang et al., 2016). There is currently no direct evidence indicating its therapeutic value for pSS and HT, which is worthy of further exploration. IGc1 is a classic Ig - like domain similar to antibody constant domains. C1 set domains are found almost exclusively in molecules involved in the immune system, such as immunoglobulin light and heavy chains, major histocompatibility complex (MHC) class I and class II complex molecules, as well as CD45^+^ T-cell receptors (Pamer and Cresswell, 1998; Radosevich and Ono, 2003). SCY is intercrineα family (small cytokine C-X-C) (chemokine CXC) involved in cell-specific chemotaxis, mediation of cell growth, and the inflammatory response (Wedemeyer et al., 2020). MHC IIα glycoproteins on antigen-presenting cells resent extracellular peptide antigens from foreign bodies such as bacteria. MHC II receptors display antigens for helper T cells and inflammatory T cells (Holling et al., 2004).

While the findings of this study are significant, there are a few limitations to consider. Firstly, the sample size was relatively small. Secondly, all the analyses were conducted *in silico*, which may not fully reflect the complexity of the biological system. To validate the predicted genes, altered immune cells, and enriched signaling pathways identified in this study, further investigation through animal and cell experiments is necessary.

## Conclusion

In summary, we comprehensively analyzed publicly available databases and gene expression microarray data from patients with pSS or HT and healthy controls. We profiled cross-talk genes and did enrichment and annotation. We identified two genes (STAT1, PTPRC) as potential biomarkers of disease activity in pSS and HT. A new understanding of the cellular and molecular mechanisms associated with pSS and HT is provided according to the immune infiltration and correlation analysis and single-gene GSEA analysis. Potential drugs and protein domains for pSS and HT are predicted based on the key genes and cross-talk genes.

## Data Availability

The original contributions presented in the study are included in the article/supplementary material, further inquiries can be directed to the corresponding authors.

## Abbreviations

AITD, autoimmune thyroid disease; ALPS, autoimmune-like lymphoproliferative syndrome; DEGs, differentially expressed genes (DEGs); GEO, Gene Expression Omnibus; GSEA, gene set enrichment analysis; HT, Hashimoto’s thyroiditis (HT); KEGG, Kyoto Encyclopedia of Genes and Genomes; MSigDB, Molecular Signatures Database; NES, normalized enrichment score; pSS, primary Sjögren’s syndrome; SS, Sjogren’s syndrome; sSS secondary Sjögren’s syndrome; TPOAbs thyroid peroxidase; Trm tissue-resident memory T.
